# Effect of Normobaric Hypoxia on Invasive Exercise Hemodynamics in Patients with Pulmonary Vascular Disease: A Randomized Controlled Trial

**DOI:** 10.1164/rccm.202409-1737RL

**Published:** 2025-01-23

**Authors:** Mona Lichtblau, Simon R. Schneider, Julian Müller, Meret Bauer, Arcangelo F. Carta, Laura Mayer, Esther I. Schwarz, Michael Furian, Stéphanie Saxer, Silvia Ulrich

**Affiliations:** Clinic of Pulmonology, Center for Pulmonary Hypertension, University Hospital Zurich, Zurich, Switzerland

*To the Editor*:

In patients with pulmonary vascular disease (PVD) classified as pulmonary arterial hypertension or chronic thromboembolic pulmonary hypertension (PAH or CTEPH), the leading symptom is dyspnea on exertion. With recent therapeutic advances, symptom burden has lessened, and patients wish to participate in a near-normal life, including travel by airplane or to the mountains. In patients with PVD, little is known about the hemodynamic adaptive changes and risks of exposure to hypoxia, especially during exercise. Current guidelines recommend that symptomatic and/or hypoxemic patients with pulmonary hypertension should avoid traveling to altitude >1,500 m (1). Hypoxic pulmonary vasoconstriction is a well-known adaptive mechanism to acute alveolar hypoxia and feared to be of particular danger to patients with PVD because of a further increase in pulmonary artery pressure (PAP) and pulmonary vascular resistance (PVR). In healthy subjects, hypobaric hypoxia increases PAP as well as PVR during acute and prolonged exposure to high altitude (2). Meanwhile, recent nonrandomized right heart catheter studies in patients with PVD showed only minor changes of pulmonary hemodynamics at rest while breathing hypoxic gas (Fi_O_2__, 0.15) (3, 4), and in patients with PVD the mean PAP (mPAP)/cardiac output (CO)-slope assessed by echocardiography did not change after short exposure to 2,500 m (5). To what extent acute normobaric hypoxia affects invasively measured exercise hemodynamics in patients with PVD is unknown. To answer the discrepancy between guideline recommendations and recent noninvasive data, we performed a single-blind, crossover, randomized placebo-controlled trial (RCT) with the gold-standard right heart catheterization to investigate the effect of hypoxia on mPAP/CO-slope and other hemodynamic parameters during exercise. Some of the results of this study have been previously reported in the form of an abstract (6).

## Methods

Patients with precapillary pulmonary hypertension (mPAP > 20 mm Hg, PVR > 2 Wood units [WU], and pulmonary artery wedge pressure ⩽15 mm Hg) classified as PAH or CTEPH were eligible to participate. Patients were excluded if they had resting hypoxemia (Pa_O_2__ < 8 kPa/60 mm Hg), were exposed to altitude >1,000 m for three or more nights within 2 weeks, or had other concomitant severe diseases.

All participants provided informed consent. The trial was registered (clinicaltrials.gov: NCT04697875) and approved by the local ethics committee (KEK Project-ID BASEC Nr. 2020-02163).

We placed a Swan-Ganz catheter (CCOmbo V, Edwards Lifesciences) via jugular vein access into the pulmonary artery and a radial artery catheter. Transducers were placed at 50% of the back-sternum distance.

Normobaric hypoxia (Fi_O_2__, 0.15; corresponding to ≈2,500 m) or placebo-ambient air (Fi_O_2__, 0.21) was administered with an altitude simulator device (AltiTrainer, SMTEC SA) through a mask in randomized sequences. Participants were connected to a breath analyzer (Ergostick, Geratherm) via a non-rebreathing two-way valve (T-shape; Hans Rudolph). CO was calculated by direct Fick using arterial and mixed venous blood gases and oxygen uptake. During each sequence, full hemodynamics were assessed in semi-supine 45° position at rest after 10 minutes resting and at the end of submaximal exercise on a cycle ergometer. The exercise duration was 6 minutes with two 3-minute steps, starting with 10–20 W, followed by 20–40 W according to the patient’s fitness. Patients rested during a washout period of >15 minutes between sequences. We calculated individual mPAP/CO-slopes as (mPAPend-exercise − mPAPrest)/(COend-exercise − COrest).

The main outcome was the difference in mPAP/CO-slope during exercise breathing normobaric hypoxia compared with placebo-air. Other outcomes were the differences in pulmonary and systemic hemodynamics, blood oxygenation, and metabolic measurements during hypoxia versus placebo-air.

A sample size of 17 patients was calculated for an assumed clinically important difference in mPAP/CO-slope of −0.7 WU (SD ± 1.0), corresponding to a power of 80% and a two-sided α of 0.05. The effect size was based on consensus among the clinical specialists.

Mixed linear regression models were applied, correcting for the intervention sequence, a time variable to test for carryover effects with a dichotomous variable for PVD-targeted pretreatment as fixed effect and subjects as random intercept effect. Statistical analysis was performed with R studio (version 4.3.2).

## Results

**Figure 1.**
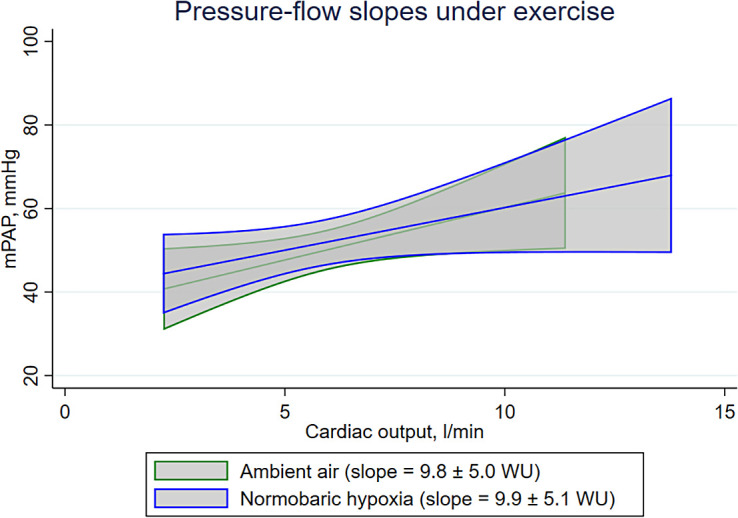
mPAP/cardiac output slopes in patients with pulmonary vascular disease assessed by right heart catheterization during exercise. The predicted regression line and 95% confidence intervals calculated from linear regression models are shown for ambient air (green) and normobaric hypoxia (blue). mPAP = mean pulmonary artery pressure; WU = Wood units.

Of 31 patients screened, 23 were included, 10 (43%) females, 15 with PAH, 8 with CTEPH, mean age 53 ± 15 years, BMI 26.0 ± 4.2 kg/m^2^, 9 incident, 14 prevalent and treated, and 10 with combination therapy. mPAP/CO-slopes were similar during placebo-air versus normobaric hypoxia (9.8 ± 5.0 WU vs. 9.9 ± 5.1 WU; mean difference [95% confidence interval] of 0.2 [−1.7 to 2.0]; *P* = 0.869) (Figure 1). Hemodynamics, blood oxygenation, and metabolic measurements at rest and end-exercise are shown in Table 1. With the exception of the expected lower blood oxygenation and an increased $\dot{\text{V}}$e/$\dot{\text{V}}$co_2_ while breathing normobaric hypoxia, no differences between placebo-air and normobaric hypoxia were detected, especially for resting hemodynamics. Exercise desaturation was more pronounced during normobaric hypoxia.

**Table 1.** Assessments at Rest and End-Exercise in Semi-supine 45° Position under Placebo-Air (Fi_O_2__, 0.21) and Normobaric Hypoxia (Fi_O_2__, 0.15)

	Measurements at Rest				Measurements at End-Exercise			
	Placebo-Air (Fi_O_2__, 0.21)	Hypoxia (Fi_O_2__, 0.15)	Mean Difference (95% CI)	*P* Value	Placebo-Air (Fi_O_2__, 0.21)	Hypoxia (Fi_O_2__, 0.15)	Mean Difference (95% CI)	*P* Value
Hemodynamics								
Heart rate, bpm	78 ± 12	79 ± 12	1 (−4 to 6)	0.683	103 ± 19	105 ± 16	2 (−3 to 8)	0.378
BP_mean_, mm Hg	92 ± 10	91 ± 10	−1 (−5 to 3)	0.553	103 ± 13	104 ± 14	1 (−3 to 5)	0.548
SVR, WU	20 ± 7.4	20.1 ± 8.4	0 (−2 to 3)	0.929	17.6 ± 5.5	18.1 ± 6.6	0.6 (−1.8 to 3.1)	0.620
PAP_mean_, mm Hg	39 ± 10	40 ± 11	1 (−3 to 4)	0.740	61 ± 16	64 ± 16	3 (−1 to 7)	0.103
PAWP, mm Hg	10 ± 3	10 ± 3	0 (−1 to 1)	0.674	12 ± 3	12 ± 3	0 (−1 to 1)	0.737
RAP, mm Hg	5 ± 3	5 ± 3	0 (−1 to 1)	0.928	9 ± 5	9 ± 5	0 (−1 to 2)	0.590
CO, L/min	4.7 ± 1.5	4.4 ± 1.6	−0.4 (−1.1 to 0.4)	0.312	7.2 ± 2.0	7.0 ± 2.8	−0.3 (−1.0 to 0.5)	0.470
PVR, WU	7.0 ± 3.8	7.6 ± 4.6	0.6 (−0.3 to 1.5)	0.175	7.5 ± 3.4	8.4 ± 4.2	0.9 (−0.5 to 2.3)	0.199
mPAP/CO-slope, WU	—	—	—	—	9.8 ± 5.0	9.9 ± 5.1	0.2 (−1.7 to 2.0)	0.869
Blood gas analyses								
pH	7.45 ± 0.04	7.46 ± 0.03	0.02 (0.0 to 0.03)	**0.008**	7.43 ± 0.03	7.44 ± 0.02	0.02 (0 to 0.03)	**0.006**
Hemoglobin, g/dl	15.2 ± 1.4	15.3 ± 1.4	0.1 (0 to 0.3)	0.167	15.6 ± 1.5	15.7 ± 1.5	0.1 (−0.1 to 0.2)	0.226
Pa_O_2__, kPa	10.2 ± 1.6	8 ± 1.5	−2.3 (−2.8 to −1.8)	**<0.001**	8.7 ± 1.6	6.3 ± 0.9	−2.4 (−2.9 to −2)	**<0.001**
Pa_CO_2__, kPa	4.1 ± 0.4	4.0 ± 0.4	−0.1 (−0.3 to 0)	0.117	4.4 ± 0.3	4.1 ± 0.3	−0.2 (−0.4 to −0.1)	**0.007**
Sa_O_2__,%	94 ± 3	91 ± 4	−4 (−6 to −2)	**<0.001**	91 ± 4	83 ± 6	−8.3 (−10.2 to −6.5)	**<0.001**
Smv_O_2__, %	69 ± 6	65 ± 7	−4 (−9 to 0)	0.064	45 ± 12	40 ± 12	−5.2 (−9.2 to −1.3)	**0.010**
Ergospirometric measurements								
$\dot{\text{V}}$o_2_, L/min/kg	3.4 ± 1.1	3.6 ± 1.7	0.1 (−2.1 to 2.2)	0.964	9.2 ± 3.4	9.6 ± 8.4	0.6 (−1.58 to 2.78)	0.586
$\dot{\text{V}}$co_2_, L/min	0.2 ± 0.1	0.3 ± 0.1	0.0 (−0.1 to 0.1)	0.817	0.6 ± 0.3	0.6 ± 0.3	0.01 (−0.06 to 0.08)	0.788
$\dot{\text{V}}$e, L/min	12.0 ± 3.1	12.9 ± 5	0.7 (−2.3 to 3.7)	0.638	27.9 ± 9.8	30.1 ± 11.7	2.4 (−0.7 to 5.4)	0.129
$\dot{\text{V}}$e/$\dot{\text{V}}$o_2_	43.6 ± 10.1	48.2 ± 15.8	4.8 (−0.5 to 10.1)	0.078	38.8 ± 11.2	44 ± 12.7	4.8 (−0.7 to 10.3)	0.084
$\dot{\text{V}}$e/$\dot{\text{V}}$co_2_	48.4 ± 7.2	49.7 ± 9.2	1.2 (−1.2 to 3.7)	0.320	42.8 ± 6.8	46 ± 6.9	3.2 (0.7 to 5.7)	**0.013**
BF, 1/min	16.3 ± 4.4	17.1 ± 4.5	1 (−0.8 to 2.7)	0.274	23.4 ± 6.4	23.6 ± 6.6	0.4 (−1.4 to 2.2)	0.670
BR, L/min	89.6 ± 2.5	88.9 ± 3.7	−0.6 (−3.2 to 2)	0.649	75.8 ± 8.3	73.6 ± 10.2	−2.2 (−4.9 to 0.4)	0.102
HRR, beat	89 ± 16	87 ± 17	0.1 (−7.1 to 7.2)	0.981	67 ± 29	59 ± 18	−5.2 (−13 to 2.6)	0.190
O_2_ pulse, ml/kg/beat	3.5 ± 1.5	3.7 ± 2.1	0.1 (−0.9 to 1.2)	0.815	6.9 ± 2.9	6.7 ± 4.6	0.1 (−1.1 to 1.3)	0.869
Pet_O_2__, kPa	13.7 ± 1.3	10.8 ± 1.3	−3 (−3.6 to −2.5)	**<0.001**	13.7 ± 1.2	10.3 ± 1.2	−3.4 (−4 to −2.9)	**<0.001**
Pet_CO_2__, kPa	3.7 ± 0.5	3.6 ± 0.5	−0.1 (−0.3 to 0.1)	0.227	3.8 ± 0.4	3.5 ± 0.4	−0.3 (−0.4 to −0.1)	**0.001**

*Definition of abbreviations*: BF = breathing frequency; BP = blood pressure; BR = breathing reserve; CI = confidence interval; CO = cardiac output; HRR = heart rate reserve; PAP = pulmonary artery pressure; PAWP = pulmonary arterial wedge pressure; Pet_CO_2__ = end-tidal carbon dioxide output; Pet_O_2__ = end-tidal oxygen uptake; PVR = pulmonary vascular resistance; RAP = right atrial pressure; Smv_O_2__ = mixed venous oxygen saturation; SVR = systemic vascular resistance; $\dot{\text{V}}$e/$\dot{\text{V}}$co_2_ = ventilatory equivalent for CO_2_; $\dot{\text{V}}$e/$\dot{\text{V}}$o_2_ = ventilator equivalent for O_2_; WU = Wood units.

Data are shown as mean ± SD and mean differences with 95% CI.

Bold text indicates statistical significance (*P* < 0.05).

## Discussion

This is the first single-blinded RCT investigating the effect of normobaric hypoxia versus placebo-air on invasive exercise hemodynamics in patients with PVD. The results show that the mPAP/CO-slope during mild to moderate exercise was not altered by hypoxia equivalent to ≈2,500 m and that neither resting nor end-exercise pulmonary hemodynamics were changed compared with placebo-air despite decreased blood oxygenation.

This study confirms previous nonrandomized, open-label studies that normobaric hypoxia does not relevantly alter invasive resting hemodynamics, including mPAP, in PVD (3, 4). This study confirms results of two studies in patients with PVD using stress-echocardiography during exercise: one RCT studying the effect of normobaric hypoxia versus placebo-air and one RCT exposing patients to hypobaric hypoxia at 2,500 m (5, 7). Both trials showed an unchanged pressure–flow relationship during exercise breathing hypoxic versus normoxic air. Together with the present RCT using invasive hemodynamics, we conclude that patients with PVD with chronic remodeling of the small pulmonary arteries reveal blunted hypoxic pulmonary vasoconstriction. Whether altered mitochondrial redox signaling or alterations in potassium or calcium channels of the smooth muscle cells of the pulmonary arteries are responsible for this effect is still unknown (8). Whether those results extend to altitudes >2,500 m and prolonged stays is unknown. It is equally unclear whether this attenuated response persists with subacute or longer exposure to hypoxia or if pulmonary hemodynamics would worsen over time in a hypoxic environment. Initial field studies in patients with PVD exposed between 5 hours and 2 days to altitude (2,048 m or 2,500 m) showed that patients subjectively tolerated the trip well, and none of the patients were symptomatic or needed evacuation, but 11–37% had severe, mainly nocturnal, asymptomatic hypoxemia at altitude requiring oxygen therapy, which promptly restored low-altitude oxygenation (9–11).

In summary, this RCT demonstrated that normobaric hypoxia versus placebo-air acutely applied during exercise right heart catheterization does not change the mPAP/CO-slope or other invasive hemodynamics at rest or mild to moderate exercise. Thus, this study may help to counsel patients with PVD who wish to visit mountain areas. However, whether mPAP/CO-slopes during exercise change upon prolonged exposure to hypoxia remains to be determined.
